# Impact of Dental Status on Heart Transplantation Outcomes

**DOI:** 10.1111/odi.70157

**Published:** 2025-11-27

**Authors:** Katharina Theresa Obermeier, Ina Dewenter, Maximilian Vorstandlechner, Maximilian Geyer, Wenko Smolka, Christine Kamla, Madeleine Orban, Christoph S. Mueller, Anusha Abdullah

**Affiliations:** ^1^ Department Oral & Maxillofacial Surgery and Facial Plastic Surgery Ludwig Maximilians University Munich Germany; ^2^ Division of Thoracic Surgery University Hospital, LMU Munich Munich Germany; ^3^ Departments of Cardiac Surgery LMU University Hospital, LMU Munich Munich Germany; ^4^ Departments of Medicine I University Hospital, LMU Munich Munich Germany; ^5^ German Centre for Cardiovascular Research (DZHK) Partner Site Munich Heart Alliance Berlin Germany

**Keywords:** dental care, heart transplantation, oral health, transplantation outcomes

## Abstract

**Objective:**

Oral infections and poor dental health may contribute to systemic complications after heart transplantation, yet data on their impact is limited. In the absence of standardized dental guidelines for transplant candidates, this study evaluated preoperative dental status and its association with post‐transplant outcomes.

**Methods:**

A retrospective analysis was conducted on 72 patients who underwent heart transplantation between 2014 and 2019. Collected data included transplantation indications, dental findings, and postoperative outcomes. All patients received a preoperative panoramic dental X‐ray and dental consultation.

**Results:**

Patients had an average of 23.88 teeth, and all presented with at least one dental filling. Periodontal abnormalities were present in 68%. While the decayed, missing, and filled teeth scores showed no significant association with post‐transplant mortality (*p* = 0.076), both periodontal disease and carious lesions were significantly associated with sepsis (*p* = 0.003). Sepsis, in turn, was significantly linked to increased mortality (*p* = 0.001).

**Conclusions:**

Poor dental health was not directly associated with post‐transplant mortality but was linked to a higher sepsis risk, which negatively affected outcomes. These findings support the need for standardized dental care protocols for heart transplant candidates.

## 1 Introduction

Heart transplantation (HTX) is a life‐saving intervention for patients with end‐stage heart failure when conventional medical and surgical therapies have failed (Lund et al. 2017). This procedure not only extends survival but also significantly improves quality of life (Starling 2010). However, HTX remains a technically demanding operation associated with numerous potential complications. The rate of HTX increased to over 3000 adult heart transplants in 2022 in the United States through the past decade, while pretransplant mortality declined (Colvin et al. 2024). Data from the International Society for Heart and Lung Transplantation (ISHLT) demonstrate a notable improvement in early outcomes, with 30‐day mortality rates decreasing from 12.1% during 1979–1985 to 6.9% in the 1996–2001 cohort (Luckraz et al. 2005). Analysis of outcomes between 1982 and 2011 demonstrates 1‐ and 5‐year survival rates of 81% and 69%, respectively, with a median survival of 11 years for the overall cohort, extending to 13 years among patients who survive beyond the first postoperative year (Lund et al. 2013). Despite these advancements, early postoperative mortality remains a significant concern, with the leading causes including primary graft failure, acute rejection, sepsis, and other postoperative complications (Jung et al. 2011).

To be listed for transplantation, candidates must meet stringent eligibility criteria, encompassing both systemic and localized health parameters (Mehra et al. 2016). Among the various factors considered in this preoperative evaluation, the patient's oral health, particularly their dental status, has emerged as a critical component (Kittleson 2025). The oral cavity represents a potential reservoir for pathogenic microorganisms, and poor dental health can lead to systemic complications (Carrizales‐Sepulveda et al. 2018), especially in immunocompromised patients following transplantation (Heimdahl and Nord 1990). Conditions such as periodontal disease, periapical abscesses, and untreated dental caries may predispose patients to severe complications (Nunes‐dos‐Santos et al. 2020). Bacteremia originating from the oral cavity can lead to severe infectious complications, most notably infective endocarditis, characterized by microbial infection of the endocardium or cardiac valves (Carinci et al. 2018). For heart transplant recipients, the risk of developing endocarditis is particularly concerning, as the use of immunosuppressive drugs significantly impairs the host immune response and makes them more susceptible to infections (Jordan et al. 2022). Data indicate that approximately 70% of patients undergoing evaluation for transplantation require oral surgical procedures for the elimination of septic foci, underscoring the substantial need for surgical dental intervention in this population (Rustemeyer and Bremerich 2007).

Thus, addressing the preoperative dental status of heart transplant candidates is a critical step in ensuring the success of the procedure and minimizing the potential for complications. The status of a heart transplant recipient's teeth and oral health is an often overlooked but critical factor in reducing the risk of complications, including graft failure. Comprehensive dental assessment and management should be an essential part of the preoperative preparation for heart transplant patients (Sezgin and Sezgin 2020). Optimizing dental health prior to transplantation might minimize the risk of infection, support the healing process, and contribute to improved postoperative recovery.

## 2 Materials and Methods

The study was approved by the Institutional Review Board of the Ludwig‐Maximilian University Hospital of Munich, Germany (Munich, Germany; Project No.: 24–0426). The present retrospective cohort study includes 72 patients who underwent HTX in our hospital during a 6‐year period from 2014 to 2019. Only patients with a preoperative panoramic radiograph, that had been conducted in our department were included, and a valid follow‐up of at least 5 years after transplantation was necessary as inclusion criteria. All demographic data, discharge summaries, survival and mortality records, as well as information regarding comorbidities, indications for transplantation, and disease progression, were retrospectively reviewed. Dental health parameters—including overall dental status, periodontal status, alveolar bone loss, and caries prevalence—were systematically assessed.

The DMFT (Decayed, Missing, and Filled Teeth) index, a standardized epidemiological tool for quantifying the prevalence and severity of dental caries in permanent dentition, was calculated to provide an objective measure of dental health.

The extent of alveolar bone loss and resorption was evaluated according to a standardized radiographic protocol described by Rydén et al. (2016). Third molars were excluded, resulting in a possible maximum of 28 teeth per patient. Dental implants were not examined. Periodontal bone loss was quantified by measuring two parameters in each tooth: the total root length (distance from the tooth apex to the cementoenamel junction) and the total bone height (distance from the tooth apex to the marginal bone crest) in each tooth. The arithmetic mean of these measurements was calculated and expressed as a percentage to represent the proportion of bone loss relative to root length (RBL).

Radiographic bone loss (RBL) was used to determine the initial stage of periodontitis according to the established classification scheme for periodontal diseases, which allows for categorization of periodontitis severity based on the extent of bone loss (Caton et al. 2018; Papapanou et al. 2018). To estimate the progression rate of periodontitis and its potential systemic impact, the grade of periodontitis was indirectly assessed by calculating the ratio of RBL to patient age at the most severely affected tooth (RBL/age). Additional risk factors, including smoking status and diabetes mellitus, were incorporated to modify the periodontitis grade.

Patients were stratified into stages based on the severity of periodontal bone loss:

Stage I (PBL < 15%), Stage II (PBL 15%–33%), and Stages III and IV (> 33%, extending to the middle or apical third of the root) (Papapanou et al. 2018). The grading of periodontitis was categorized as follows: Grade A (RBL/age < 0.25), indicating a slow rate of progression, Grade B (RBL/age between 0.25 and 1.0), indicating moderate progression, and Grade C (RBL/age > 1.0), indicating rapid progression (Papapanou et al. 2018).

For data analysis, all patients were considered for the survival analysis. Censoring was done automatically by performing statistical analysis.

### 2.1 Definition of Causes of Death

Causes of death were classified based on the final clinical diagnosis documented in the hospital discharge summary and/or the transplant follow‐up records. Sepsis was defined as a life‐threatening organ dysfunction caused by a dysregulated host response to infection, according to Sepsis‐3 criteria (Singer et al. 2016). Pneumonia was recorded when radiographic and microbiological evidence of pulmonary infection was present. Multiorgan failure (MOF) was defined as failure of at least two organ systems, usually as a terminal event, and was assigned as a primary cause of death only when no specific infectious or rejection‐related trigger was identifiable. In cases where MOF occurred secondary to sepsis or pneumonia, the underlying infectious process was recorded as the primary cause.

### 2.2 Statistical Analysis

Statistical analysis was conducted using SPSS 24 version 4.0 (SPSS Inc., Chicago, IL, USA). Thus, the data shown in figures and tables are descriptive; mean values and standard deviation were calculated. To compare all groups statistically the Mann–Whitney *U*‐test and the Chi‐Square test were performed. Statistical significance was defined as *p* < 0.05. Comparison of mean values of two groups was done by performing the t‐test. ANOVA with Bonferroni post hoc (Tukey) test was used for comparing multiple mean values. The influence of the underlying diagnosis leading to TX, patients' gender, age at TX, caries, periodontal status, and bone loss was examined. To identify the strongest risk factors for a deteriorated rate of survival and subsequently patients' death among those listed above, we used a logistic regression model and chi‐square test.

### 2.3 Statistical Power Analysis

To ensure adequate statistical power for detecting meaningful associations between dental status, periodontal health, and post‐transplant survival, we conducted an a priori power analysis for logistic regression. We assumed a medium effect size (Cohen's *h* = 0.5, approximately equivalent to an odds ratio of 2.0), a significance level of *α* = 0.05, and two main predictors: dental status (e.g., DMFT index) and periodontal status (e.g., PA grading or stage).

Based on these assumptions, a total sample size of approximately 63 patients would be required to achieve a power of 0.80. Our dataset included 72 patients with complete survival outcome data, yielding an estimated statistical power of 0.96. Thus, the available sample provides sufficient power to detect moderate effects in the association between oral health parameters and transplant survival.

## 3 Results

### 3.1 Study Population

**FIGURE 1:**
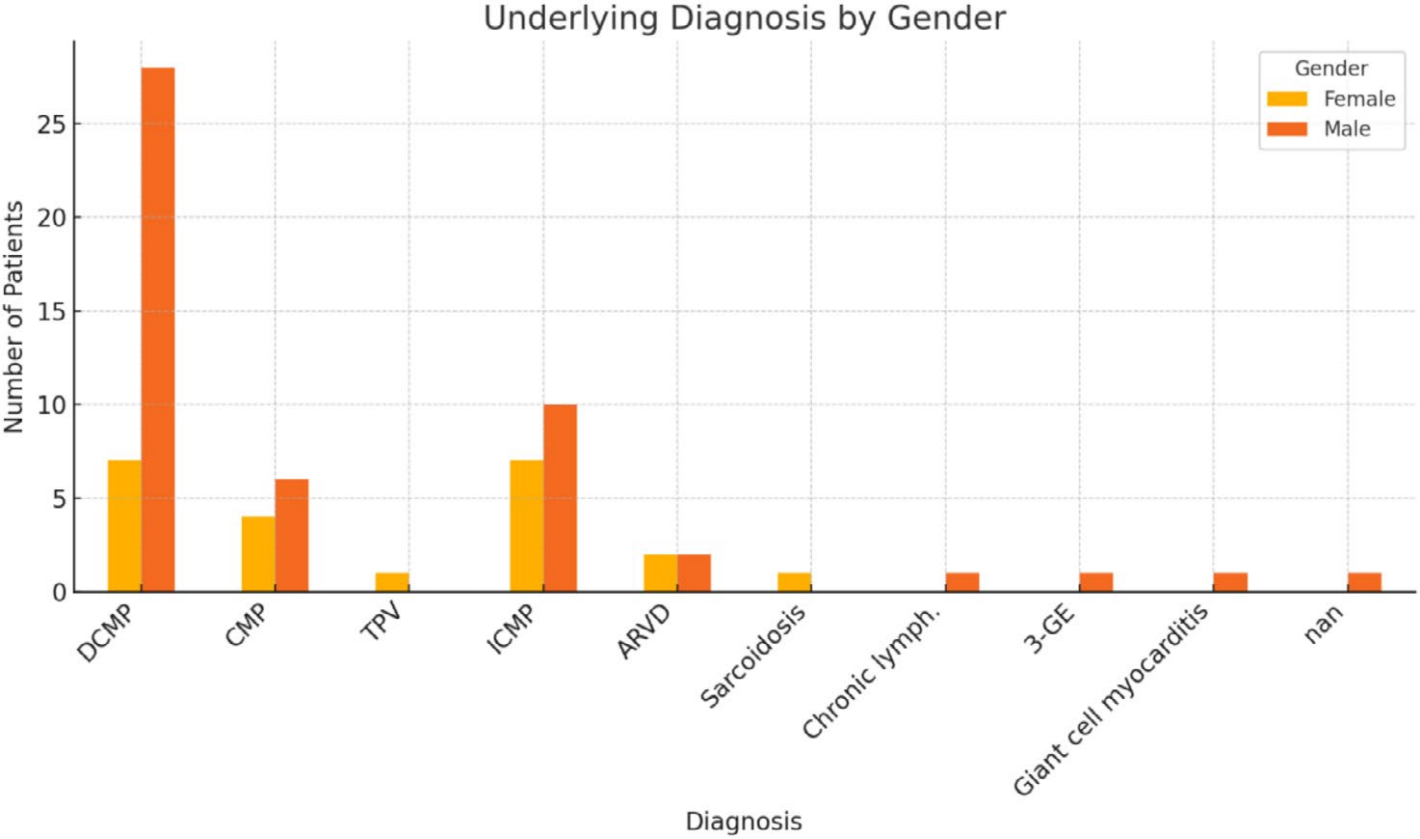
The most prevalent underlying diagnoses for HTX, stratified by gender. Dilated cardiomyopathy (DCMP) was the most common underlying diagnosis leading to HTX overall. Among male recipients, DCMP was followed by ischemic cardiomyopathy (ICMP) as the second most prevalent cause. In female recipients, both DCMP and ICMP were observed with similar frequency as leading indications for transplantation. Less common underlying conditions in both genders included other forms of cardiomyopathy (CMP), valvular heart disease (TPV), arrhythmogenic right ventricular cardiomyopathy (ARVC), sarcoidosis, chronic lymphocytic myocarditis, giant cell myocarditis (GCM), and unclassified (nan) causes.

A total of 72 patients were included in this study. Of these, 22 patients (30.5%) were female and 50 patients (69.4%) were male. The mean age at the time of transplantation was 48.75 ± 12.7 years, with a range of 18 to 63 years. Figure 1 shows the underlying diagnosis for heart transplantation. One patient received a second heart transplant. 27 patients (37.5%) reported a history of nicotine abuse. No patient reported a history of hepatitis.

**FIGURE 2:**
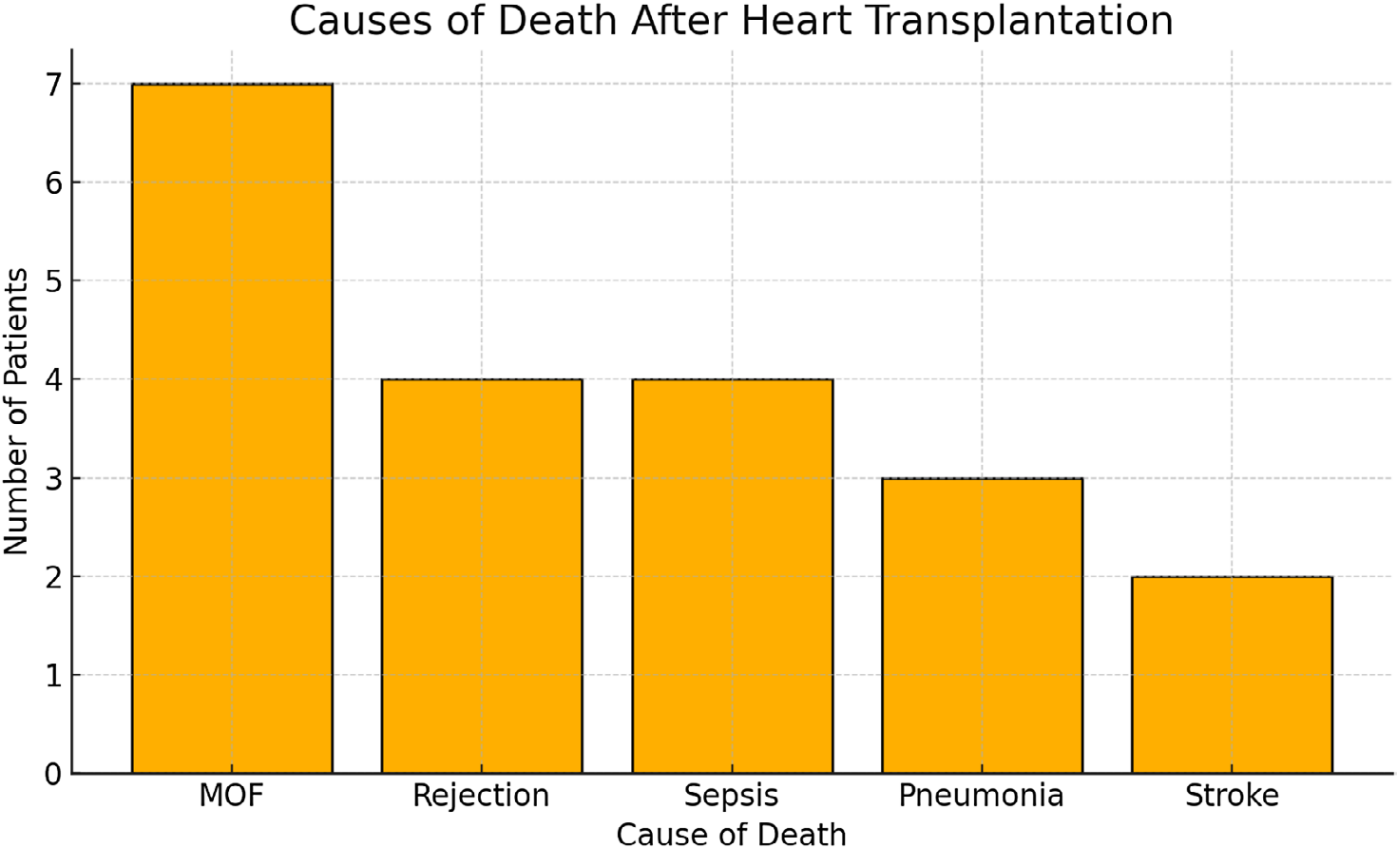
Causes of death after heart transplantation. Causes of death were classified according to the clinical and microbiological documentation as described in Section 2.1. Sepsis was defined following the Sepsis‐3 criteria (Singer et al. 2016). MOF was recorded as the primary cause only when no infectious or rejection‐related trigger was identified; when MOF occurred secondary to sepsis or pneumonia, the underlying infection was assigned as the main cause. The most frequent primary causes included sepsis, pneumonia, MOF (non‐infectious), graft rejection, and stroke.

To prevent graft failure, all patients received a combination of immunosuppressive agents, including mycophenolate mofetil, tacrolimus, and everolimus, the latter of which was introduced later in the post‐transplantation period. Prednisolone was administered as part of the post‐transplant immunosuppressive regimen. The mean follow‐up period was 1753.2 ± 1198.4 days, ranging from 1 day to almost 11 years. Following transplantation, all patients were initially admitted to the intensive care unit (ICU), where they underwent mechanical ventilation weaning and extubation. Once clinically stable, they were transferred to the general ward for ongoing postoperative care. Overall, 20 patients (27.7%) died during follow‐up, and the average time until death amounted to 325 ± 527 days. Causes of death were determined according to predefined diagnostic criteria (see Section 2.1) and are summarized in Figure 2.

### 3.2 Teeth Condition

Only patients with available preoperative panoramic dental radiographs were included in the analysis. The mean interval between the panoramic radiograph and heart transplantation was 377.68 (range 7–2316 days).

Preoperatively, patients had a mean of 23.88 ± 7.31 teeth (range from 0 to 32). Periodontal status was also assessed, with 68% of patients exhibiting periodontal abnormalities. The mean total alveolar bone height was 7.70 ± 5.79 mm, ranging from 0 to 16.60 mm. The mean TBH/TRL ratio in this study was 0.66 ± 0.13, indicating moderate periodontal bone loss within the patient cohort. The mean bone loss coefficient value of 0.425 (SD ±0.13) indicated a moderate to advanced level of periodontal bone loss across the study population, with considerable variation between individuals.

In total, 67 patients (93%) presented with at least one dental filling, with a mean of 10.00 ± 5.77 affected teeth per patient (range from 0 to 25).

The number of tooth extractions before HTX varied among 4.92 ± 6.89 tooth extractions per patient.

### 3.3 Survival and Complications

Statistically, we correlated the DMFT score with mortality after transplantation (*p* = 0.076) (Table 1). There was a weak positive correlation: higher DMFT scores are slightly associated with higher mortality. However, the *p*‐value is above 0.05, indicating that this result is not statistically significant at the conventional threshold. Additionally, no significant correlation was found between the number of periodontal sites or the presence of periodontal disease and post‐transplant mortality.

**TABLE 1:** Statistical analysis and classification of causes of death.

Variable	Outcome	Correlation	*p*
DMFT	Death	0.211	0.0757
Number of periodontal sites	Death	0.178	0.1352
PA problem	Death	0.028	0.8177
Number of periodontal sites	Sepsis	0.343	0.0032
Sepsis	Death	0.378	0.0011
Number of carious lesions	Sepsis	0.343	0.0032

*Note:* Causes of death were classified as described in Section 2.1. Sepsis was defined according to the Sepsis‐3 criteria (Singer et al. 2016). MOF was recorded as the primary cause only when no infectious or rejection‐related trigger was identified; MOF secondary to infection was assigned to the corresponding underlying cause (e.g., sepsis or pneumonia).

In contrast, a statistically significant positive correlation was identified between the number of periodontal sites and the incidence of sepsis (*p* = 0.003). Sepsis itself was significantly associated with increased post‐transplant mortality (*p* = 0.001). Moreover, a moderate positive correlation was observed between the number of carious lesions and the occurrence of sepsis (*p* = 0.003).

Among heart transplant recipients who developed sepsis (*n* = 4), a significant correlation was observed between poor dental status—particularly the presence of periodontitis and carious lesions—and the incidence of septic complications (*p* = 0.0032). However, microbiological and clinical evaluation identified alternative infectious sources in several cases. Three patients had pneumonia, including two with confirmed Aspergillus pneumonia and one with COVID‐19 pneumonia. One patient had a bloodstream infection with coagulase‐negative Staphylococcus. Another patient had *Klebsiella pneumoniae* detected in a throat swab, consistent with respiratory tract colonization or infection.

These findings indicate that in a proportion of cases, sepsis was associated with non‐oral infectious foci, and a direct contribution of dental status in these specific patients cannot be confirmed.

**FIGURE 3:**
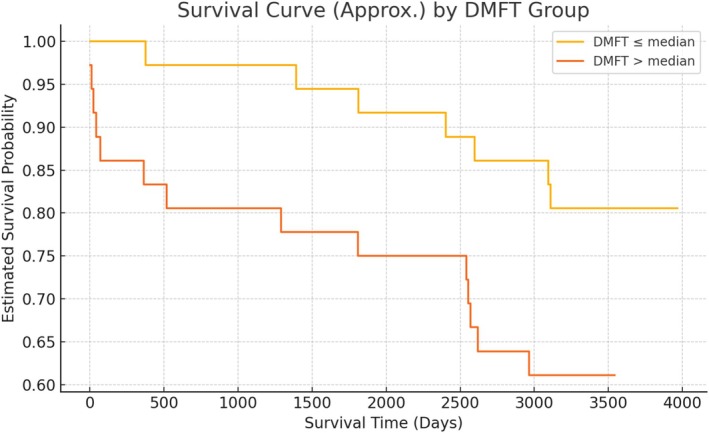
Kaplan–Meier survival curves by DMFT group. Kaplan–Meier survival analysis illustrating post‐transplant survival over a 10‐year follow‐up period, comparing patients with high DMFT scores (DMFT > median) to those with low/moderate scores (DMFT≤median). Patients with higher DMFT scores exhibited significantly reduced survival compared to those with lower scores (log‐rank test).

**FIGURE 4:**
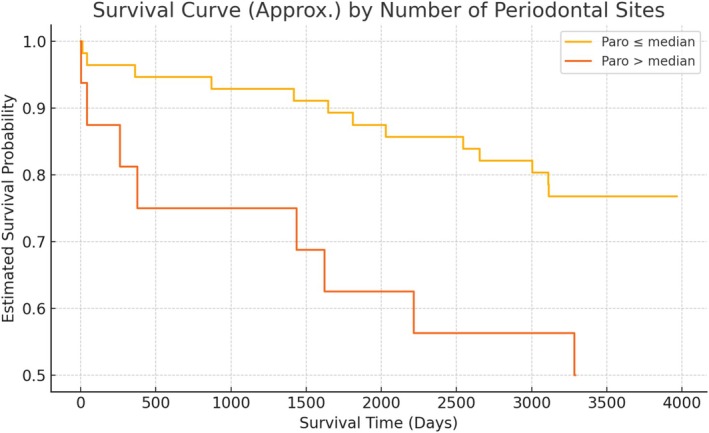
Kaplan–Meier survival curves by the number of periodontal sites. Kaplan–Meier survival analysis illustrating post‐transplant survival over a 10‐year follow‐up period, comparing patients with a high versus low number of periodontal sites (dichotomized at the median). Patients with a higher number of periodontal sites showed significantly reduced survival compared to those with fewer sites (log‐rank test).

**FIGURE 5:**
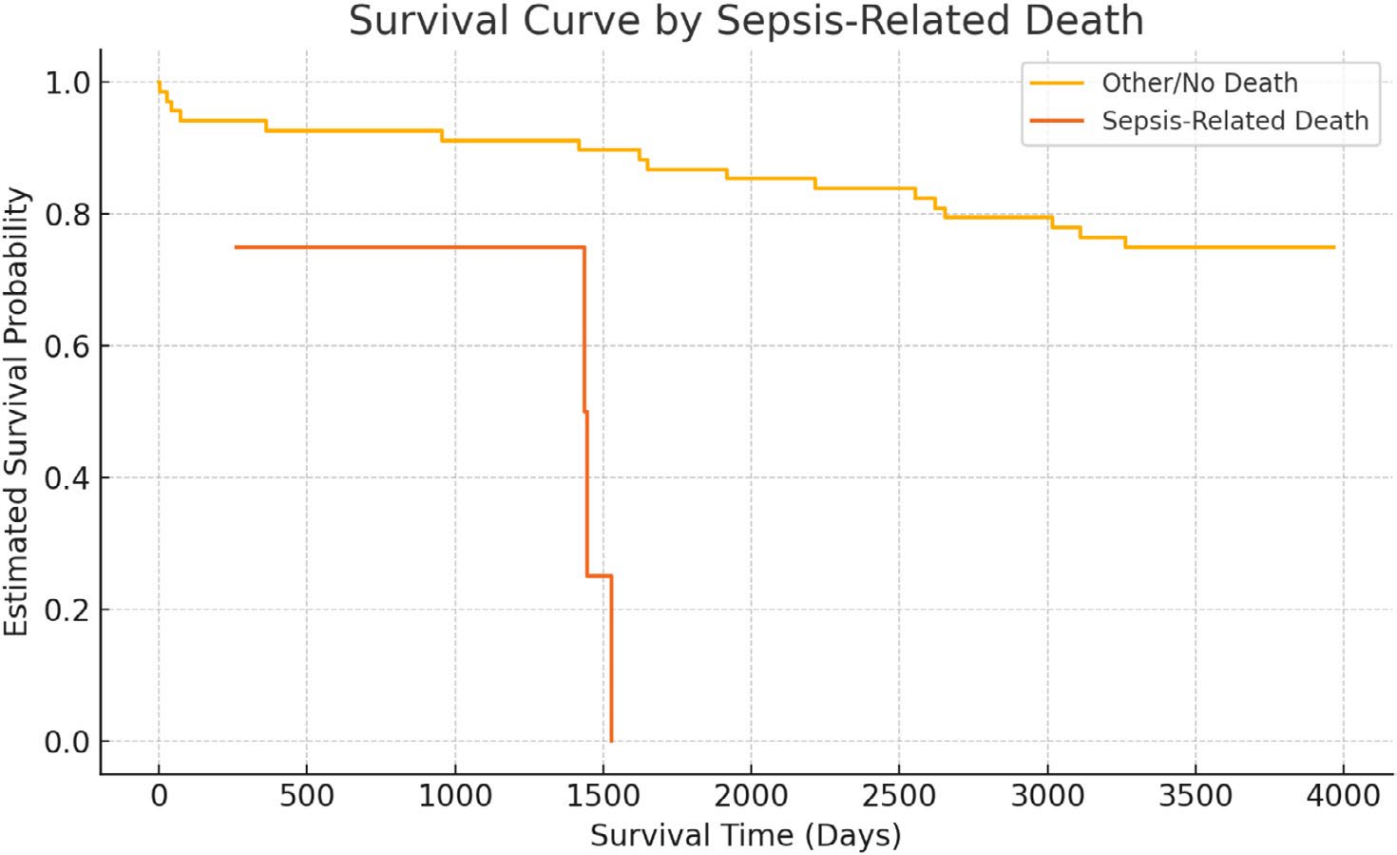
Kaplan–Meier survival curves by cause of death. Kaplan–Meier survival analysis showing post‐transplant survival in patients with death related to sepsis versus no death or death due to other causes. The analysis covers a 10‐year follow‐up period. Patients who died due to sepsis showed significantly reduced survival times compared to those with other causes of death or no death (log‐rank test).

There is a weak negative correlation between the number of teeth and death: patients with fewer teeth tended to have higher mortality. However, the result is not statistically significant (*p* > 0.05; Figures 3, 4, 5).

## 4 Discussion

Existing literature indicates that only minimal attention has been given to examining dental health, oral conditions, and their potential impact on post‐transplant outcomes in this patient population. To date, no studies have specifically explored the relationship between preoperative dental status and periodontal health and postoperative survival following HTX.

The present study highlights a considerable burden of oral disease among patients undergoing HTX, as evidenced by the high prevalence of dental restorations, periodontal abnormalities, and tooth loss. All patients included in the analysis had at least one dental filling, with a mean of 10 affected teeth per patient, reflecting a significant history of dental caries and restorative treatment. Moreover, nearly 70% of patients exhibited signs of periodontal disease, consistent with findings in other chronically ill populations, where systemic inflammation and poor oral hygiene are commonly observed (Binner et al. 2019).

No significant relationship was identified between periodontal disease markers, such as the number of affected periodontal sites, and mortality post‐transplantation. This contrasts with some reports that implicate periodontal inflammation as a risk factor for systemic complications, including cardiovascular events and infection‐related morbidity in immunocompromised patients (Hajishengallis and Chavakis 2021; Tonetti and Van Dyke 2013). In transplant patients, chronic periodontitis has also been associated with elevated systemic interleukin‐6 levels, reflecting local periodontal production and suggesting a contribution to systemic inflammation (“Periodontal disease can affect organ transplant survival,” 2006). In addition, recent evidence suggests that parameters of periodontal inflammation—specifically bleeding on probing (BOP) and periodontal inflamed surface area (PISA)—may be linked to an increased risk of cardiac and systemic infections in patients with heart failure or following heart transplantation (Schmalz et al. 2023). However, our data did reveal a significant positive correlation between the number of periodontal sites and the incidence of sepsis, highlighting the potential role of periodontal pathology as a nidus for systemic infection in this vulnerable population.

Importantly, sepsis was significantly associated with increased post‐transplant mortality, underscoring its critical impact on patient outcomes. Additionally, the moderate positive correlation between carious lesions and sepsis incidence suggests that untreated dental decay may contribute to systemic infectious complications, possibly by serving as a source of bacteremia or inflammatory burden (Evans 2006; Hajishengallis and Chavakis 2021). A survey of dental care protocols among U.S. organ transplant centers reported that post‐transplantation sepsis from a suspected dental source was acknowledged in 27% of the responses; however, the type of transplanted organ involved was not specified (Guggenheimer et al. 2005). Nevertheless, and in agreement with our findings, these data underline a potential correlation between poor dental status and post‐transplant sepsis.

At the same time, microbiological analyses in our cohort revealed that several septic episodes were associated with infectious foci outside the oral cavity, including *Aspergillus* and COVID‐19 pneumonia as well as bloodstream infection. These observations indicate that, while oral health may be relevant in the context of sepsis risk, non‐oral infections also represent important contributors in this highly immunocompromised population. This supports the need for a comprehensive infection prevention and surveillance strategy in post‐transplant care, extending beyond pre‐transplant dental optimization.

It is also important to note that, to date, no official guidelines exist concerning the management of oral health in heart transplant candidates. There are no standardized protocols for pre‐transplant dental evaluation or treatment, resulting in considerable variability in clinical practice. In the present study, data were collected from a specialized transplant center where dental assessments are routinely performed prior to transplantation. As a result, necessary dental interventions—such as restorations, root canal treatments, and tooth extractions—can be performed prior to transplantation, thereby reducing potential sources of oral inflammation. Evidence from several studies indicates gaps in the dental management of organ transplant recipients (Kwak et al. 2020; Ziebolz et al. 2011). In the absence of such preemptive care, oral health status in this population might be significantly worse, which, as suggested by studies in other organ transplant recipients, could adversely impact post‐transplant outcomes (Helenius‐Hietala et al. 2013) (Olsson et al. 2024).

Due to the retrospective design, detailed periodontal parameters such as clinical attachment loss were not consistently available, limiting the precision of periodontal disease classification. Carious lesions were diagnosed from dental radiographs rather than clinical examination, which may have led to underestimation or misclassification of decay severity.

Because of its observational nature, the study could not control for potential confounding factors, including variations in immunosuppressive therapy, patient comorbidities, and infection control practices, which limit the ability to draw causal conclusions. While our findings suggest an association between periodontal status and post‐transplant sepsis, causality cannot be established due to the retrospective nature of the analysis.

## 5 Conclusion

In conclusion, this study reveals notable deficits in dental health among patients awaiting HTX. Our findings demonstrate a correlation between the severity of periodontitis and the incidence of sepsis. Additionally, a significant association was observed between sepsis and post‐transplant mortality. However, no direct correlation could be established between pre‐transplant dental conditions and post‐transplant mortality. These results highlight the potential mediating role of systemic infections in linking oral health to transplant outcomes and underscore the need for further investigation.

These findings emphasize the critical role of pre‐transplant dental screening and intervention. Comprehensive dental assessments, including panoramic radiographs, allow for the identification and management of oral pathologies that may pose a risk to transplant success. The integration of dental professionals into the transplant team is therefore essential for optimizing outcomes and reducing postoperative morbidity.

To strengthen these observations, future research should focus on prospective studies with larger sample sizes, ideally conducted across multiple centers to increase generalizability. Longitudinal studies are particularly needed to assess whether targeted pre‐transplant dental interventions can lower sepsis rates and improve long‐term survival. Incorporating structured dental care into transplant protocols may represent a valuable strategy for improving clinical outcomes in this vulnerable population.

## Author Contributions

**Katharina Theresa Obermeier:** conceptualization, methodology, investigation, formal analysis, supervision, writing – original draft, writing – review and editing, visualization. **Ina Dewenter:** conceptualization, investigation. **Maximilian Vorstandlechner:** validation, methodology, writing – review and editing. **Maximilian Geyer:** investigation, formal analysis. **Wenko Smolka:** conceptualization, formal analysis, writing – review and editing. **Christine Kamla:** formal analysis, investigation. **Madeleine Orban:** methodology, validation. **Christoph S. Mueller:** validation, methodology, writing – review and editing. **Anusha Abdullah:** conceptualization, investigation, writing – original draft, writing – review and editing, formal analysis, software, methodology, visualization.

## Conflicts of Interest

The authors declare no conflicts of interest.

## Data Availability

The datasets generated and/or analyzed during the current study are not publicly available due to the anonymization process, but are available from the corresponding author on reasonable request.
